# Imminence of death among hospital inpatients: Prevalent cohort study

**DOI:** 10.1177/0269216314526443

**Published:** 2014-03-17

**Authors:** David Clark, Matthew Armstrong, Ananda Allan, Fiona Graham, Andrew Carnon, Christopher Isles

**Affiliations:** 1 School of Interdisciplinary Studies, University of Glasgow, Dumfries, UK; 2 Healthcare Information Group, Information Services Division, NHS National Services Scotland, Edinburgh, UK; 3 Department of Public Health, NHS Dumfries & Galloway, Dumfries, UK; 4 Glenkens Medical Practice, New Galloway, UK; 5 Dumfries and Galloway Royal Infirmary, NHS Dumfries & Galloway, Dumfries, UK

**Keywords:** Inpatients, hospitalisation, palliative care, terminal care, end-of-life care, social deprivation, mortality

## Abstract

**Background:**

There is a dearth of evidence on the proportion of the hospital population at any one time, that is in the last year of life, and therefore on how hospital policies and services can be oriented to their needs.

**Aim:**

To establish the likelihood of death within 12 months of a cohort of hospital inpatients on a given census date.

**Design:**

Prevalent cohort study.

**Participants:**

In total, 10,743 inpatients in 25 Scottish teaching and general hospitals on 31 March 2010.

**Results:**

In all, 3098 (28.8%) patients died during follow-up: 2.9% by 7 days, 8.9% by 30 days, 16.0% by 3 months, 21.2% by 6 months, 25.5% by 9 months and 28.8% by 12 months. Deaths during the index admission accounted for 32.3% of all deaths during the follow-up year. Mortality rose steeply with age and was three times higher at 1 year for patients aged 85 years and over compared to those who were under 60 years (45.6% vs 13.1%; *p* < 0.001). In multivariate analyses, men were more likely to die than women (odds ratio: 1.18, 95% confidence interval: 0.95–1.47) as were older patients (odds ratio: 4.99, 95% confidence interval: 3.94–6.33 for those who were 85 years and over compared to those who were under 60 years), deprived patients (odds ratio: 1.17, 95% confidence interval: 1.01–1.35 for most deprived compared to least deprived quintile) and those admitted to a medical specialty (odds ratio: 3.13, 95% confidence interval: 2.48–4.00 compared to surgical patients).

**Conclusion:**

Large numbers of hospital inpatients have entered the last year of their lives. Such data could assist in advocacy for these patients and should influence end-of-life care strategies in hospital.

**What is already known about this topic?**

- Understanding of the high proportion of deaths occurring in hospital is well established.
- Some data exist on the proportion of patients in hospital likely to benefit from palliative care.
- There is evidence of difficulty in making the transition to palliative care for patients in hospital.

**What this study adds?**

- This is the first study of its kind to establish the proportion of hospital inpatients who die over a period of 12 months from a given date.
- The study shows how the likelihood of death in 12 months is related to male gender, age, admission to a medical specialty and social deprivation.

**Implications for practice, theory or policy**

- In order that appropriate care plans can be made and delivered for patients, there is a strong need for hospitals to adopt a more vigorous approach to identify patients who are entering the last year of their lives.
- We contend that the culture and organisation of hospitals need to become more attuned to the high proportion of inpatients in imminent need of end-of-life care.

## Introduction

There is growing interest in the challenge of providing appropriate end-of-life care to an ageing population, as demands on services increase and as expectations of patients and families change.^1,2^ The role of the hospital in the delivery and planning of such care is of major significance, partly because so many patients die there, but also because hospital admission provides an opportunity to identify those who may be approaching death.^3,4^ The likelihood of dying in hospital varies across countries, but is generally high. One recent study involving 36 national jurisdictions ranked Scotland 12th from the top for the proportion of all deaths occurring in hospital (59%); Japan (78%) had the highest proportion of hospital deaths.^5^ A study of six European countries found significant national variation in the proportions of all deaths that occurred in hospital, from 33.9% (Netherlands) to 62.8% (Wales).^6^ Older people are those most likely to die in hospital. The European study showed that in Scotland, among those aged 80–84 years, 62.3% of all deaths were in hospital. In England, the greatest proportion of hospital deaths is in the 65–84 years group, at 61% of all deaths for this group.^7^

A 2001 study in one hospital in England found that 23% of the total inpatient population was identified by staff as having palliative care needs and/or being terminally ill, and 11% were considered suitable for referral to a specialist palliative care bed.^8^ A more recent study in two English hospitals using case notes to examine for evidence of palliative care need according to Gold Standards Framework (GSF) prognostic indicator criteria and including the views of medical and nursing staff and patients (or consultees) found that 36.0% of patients were identified as having palliative care needs.^9^ In an Australian hospital network, 35% of acute inpatients were identified as having palliation as the goal for their long-term care.^10^ In a group of 14 Belgian hospitals, 9.4% of inpatients were identified as ‘palliative’ by the medical and nursing staff.^11^

Such studies are useful in establishing two factors: the propensity to die in hospital and the proportion of patients in hospital at any one time who may have palliative care needs. A third perspective can be contributed by assessing the proportions and characteristics of those in hospital who are nearing the end of life and using that to shape packages of care for those patients. Knowing more about this group would create greater possibilities for advance care planning for groups of patients, even if individual prognostication is problematic.^12,13^ In an acute hospital in New Zealand, it was found that 19.8% of inpatients included in a census met at least one of the GSF prognostic indicators, suggesting that they were likely to be in the last year of life;^14^ but it is not known if the prognostication proved accurate. We set out to answer two questions in the Scottish context. First, what proportion of inpatients in Scotland’s teaching and general hospitals on a given date will die during the index admission and 3 months, 6 months, 9 months and 12 months later? Second, how does the proportion vary by age, gender, specialty and deprivation score?

### Participants and setting

We chose those hospitals in Scotland in which most acute clinical activity occurs, the teaching (*n* = 7) and large general hospitals (*n* = 18), as agreed by others. We established how many inpatients, excluding geriatric long stay, were in these hospitals on the census date of 31 March 2010. Teaching hospitals accounted for 4829 patients; general hospitals for 5914. A patient was counted as being in hospital overnight on 31 March 2010 if they had a Scottish Morbidity Record Scheme 01 (SMR01) episode where the admission date was 31 March 2010 or earlier and where the discharge date was 1 April 2010 or later. The source of the hospital data was the national SMR01, which records all inpatient and day case discharges from non-obstetric and non-psychiatric specialties in National Health Service (NHS) hospitals in Scotland.

## Methods

We provided statistical summaries for all data using numbers and percentages of deaths at 7 and 30 days, 3 months, 6 months, 9 months and 1 year from the census date. We used multivariate logistic regression models, using R 3.0.1, to determine whether there was any association between potential predictor variables and mortality at 1 year, adjusting for the possible confounding effect of age, gender, a measure of deprivation and whether admission was to a medical or surgical specialty. A univariate logistic regression analysis, also using R 3.0.1, was undertaken to examine the relationship between age and mortality. The measure of deprivation used here is the Scottish Index of Multiple Deprivation 2009 (SIMD09), an area-based deprivation score which groups the Scottish population into five equal quintiles, with quintile 1 representing the 20% most deprived areas in Scotland and quintile 5 the least deprived.^15^ The National Records of Scotland office provided information on deaths including the date of death.^16^

## Results

We identified 10,743 hospital inpatients on the census date. More were women (54.7%) than men (45.3%). Most (64.1%) were aged 65 years or older. A disproportionate number of admissions belonged to the two most deprived quintiles (50.1%), and more patients had been admitted to a medical (63.1%) than to a surgical specialty (36.8%).

Table 1 shows that 2.9% had died within 7 days of the census date, 8.9% by 30 days, 16.0% by 3 months, 21.2% by 6 months, 25.5% by 9 months and 28.8% by 12 months.

**Table 1.** Number and percentage of patients in teaching and general hospitals in Scotland on 31 March 2010, dying at intervals after census date.

	In hospital on 30 March 2010, *n* (%)	Deaths within 7 days, *n* (%)	Deaths within 30 days, *n* (%)	Deaths within 3 months, *n* (%)	Deaths within 6 months, *n* (%)	Deaths within 9 months, *n* (%)	Deaths within 1 year, *n* (%)	Death after 30 days (%) of all deaths within 1 year
Gender								
Men	4866 (45.3)	147 (3.0)	458 (9.4)	819 (16.8)	1095 (22.5)	1299 (26.7)	1480 (30.4)	69.1
Women	5877 (54.7)	167 (2.8)	493 (8.4)	897 (15.3)	1183 (20.1)	1439 (24.5)	1618 (27.5)	69.5
Age (years)								
Under 60	3008 (28.0)	30 (1.0)	110 (3.7)	207 (6.9)	283 (9.4)	342 (11.4)	394 (13.1)	72.1
60–64	845 (7.9)	18 (2.1)	60 (7.1)	95 (11.2)	134 (15.9)	167 (19.8)	191 (22.6)	68.6
65–69	1021 (9.5)	38 (3.7)	87 (8.5)	155 (15.2)	205 (20.1)	256 (25.1)	299 (29.3)	70.9
70–74	1185 (11.0)	35 (3.0)	105 (8.9)	191 (16.1)	267 (22.5)	314 (26.5)	355 (30.0)	70.4
75–79	1487 (13.8)	43 (2.9)	148 (10.0)	285 (19.2)	370 (24.9)	437 (29.4)	491 (33.0)	69.9
80–84	1430 (13.3)	63 (4.4)	180 (12.6)	319 (22.3)	412 (28.8)	496 (34.7)	562 (39.3)	68.0
85 and over	1767 (16.4)	87 (4.9)	261 (14.8)	464 (26.3)	607 (34.4)	726 (41.1)	806 (45.6)	67.6
Deprivation (SIMD09)								
Q1 (most)	2936 (27.3)	83 (2.8)	265 (9.0)	474 (16.1)	634 (21.6)	768 (26.2)	874 (29.8)	69.7
Q2	2453 (22.8)	79 (3.2)	230 (9.4)	395 (16.1)	526 (21.4)	629 (25.6)	720 (29.4)	68.1
Q3	1988 (18.5)	53 (2.7)	169 (8.5)	317 (15.9)	425 (21.4)	510 (25.7)	570 (28.7)	70.4
Q4	1800 (16.8)	55 (3.1)	161 (8.9)	304 (16.9)	385 (21.4)	456 (25.3)	504 (28.0)	68.1
Q5 (least)	1515 (14.1)	43 (2.8)	124 (8.2)	222 (14.7)	303 (20.0)	370 (24.4)	424 (28.0)	70.8
Specialty								
Medical	6779 (63.1)	253 (3.7)	757 (11.2)	1357 (20.0)	1793 (26.4)	2134 (31.5)	2410 (35.6)	68.6
Surgical	3954 (36.8)	61 (1.5)	194 (4.9)	359 (9.1)	485 (12.3)	603 (15.2)	686 (17.3)	71.7
Outcome of index admission								
Discharged	9742 (90.7)							
Death	1001 (9.3)							
Hospital type								
Teaching (*n* = 7)	4829 (45.0)							
Large general hospital (*n* = 18)	5914 (55.0)							
Total	10,743 (100)	314 (2.9)	951 (8.9)	1716 (16.0)	2278 (21.2)	2738 (25.5)	3098 (28.8)	69.3

SIMD09: Scottish Index of Multiple Deprivation 2009.

**Figure 1.**
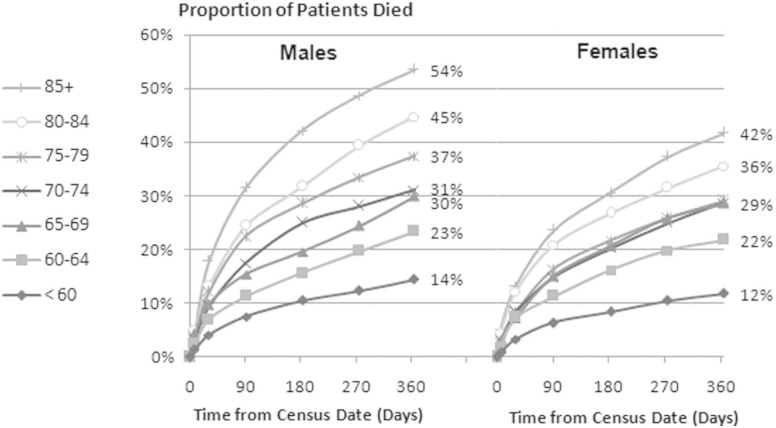
Mortality over time for all patients in teaching and general hospitals in Scotland on 31 March 2010.

Descriptive analysis showed that men were more likely to die than women for the majority of age groups at each follow-up time interval (Table 1 and Figure 1). The mortality rate rose steeply with age and was three times higher at 1 year for patients aged 85 years and over compared to those who were under 60 years of age (45.6% vs 13.1%; *p* < 0.001) (Table 1).

The univariate logistic regression showed that every 1 year increase in age at the census date was associated with a 1.04 fold increase in the mortality odds.

In multivariate analyses, men were more likely to die than women (odds ratio (OR): 1.18, 95% confidence interval (CI): 0.95–1.47) as were older patients (OR: 4.99, 95% CI: 3.94–6.33 for those who were 85 years and over compared to those who were under 60 years), deprived patients (OR: 1.17, 95% CI: 1.01–1.35 for most deprived compared to least deprived quintile) and those admitted to a medical specialty (OR: 3.13, 95% CI: 2.48–4.00 compared to surgical patients).

Of the patients, 9.3% died during the index admission, and this accounted for 1001 (32.3%) of all 3098 deaths within the 12-month follow-up period. Around 70% of the deaths (2147 of 3098) occurred more than 30 days after the census date. The 2097 patients who survived the index admission but died within 12 months of the census date required a further 4231 hospital stays (subsequent to the index stay) between 31 March 2010 and their respective dates of death.

## Discussion

Our study quantifies the large number of hospital patients who are within the last year of life and produces findings relevant to health-care priority setting. The likelihood of death during the 12 months after our census date was more closely related to age and admission to a medical specialty than to male gender and social deprivation. We have also shown that most of the deaths occur after discharge from hospital and not during the index admission.

Despite the difficulty of prognosticating for individual patients, the scale of the issue revealed here requires a response from both policymakers and clinicians. Our findings support the various initiatives currently underway to raise the profile of end-of-life care in the hospital. Best known of these, and currently the subject of much debate, is the Liverpool Care Pathway (LCP);^17^ whatever the outcome of decisions about what should replace the LCP,^18–20^ the need is clear for some structured approach to the identification and care of patients in hospital in the last days of life – shown in our data to be 9% of the hospital population. In Ireland, a wider ‘systems approach’ has been developed known as the Hospice Friendly Hospitals Programme, which seeks to promote improved end-of-life care as part of the ‘core business’ of the acute hospital.^21^ Advance Care Planning with hospital patients likewise seeks to promote good end-of-life care at a point where patients become incapable of participating in medical treatment decisions.^22^ Such interventions would benefit from more detailed knowledge of the imminence of death in the hospital population, as described here.

A recent study has highlighted the mismatch between current best practice recommendations on transitions to palliative care in acute hospitals and the observed clinical reality.^23^ Two key barriers were identified: (1) the internal momentum of the hospital towards cure inhibited clinicians from standing back and thinking about the overall goals that should inform patient care and (2) decision-making was consultant led, with junior members of the team and particularly nursing colleagues much less involved in discussion about the goals of care. Our data help inform how the approach to such issues might be targeted in the first instance.

The current UK General Medical Council guidance on end-of-life care requires doctors to ensure that death becomes an explicit discussion point when patients are likely to die within 12 months and places a strong emphasis on patient choice rather than ‘medical paternalism … however benignly intended’.^24^ The 2010 document *The Route to Success in End of Life Care – Achieving Quality in Acute Hospitals*, produced by the National End of Life Care Programme for England, makes a particular point of addressing not only a clinical audience but also board members and senior managers, indicating the need for ‘a commitment to support and review end of life care services’.^25^ This study supports clinicians and managers to give greater priority to the identification of patients at the end of life and to encourage a more proactive response to their needs.

### Strengths and limitations of the study

We are not aware of another study of this type, which requires sophisticated techniques of record linkage that connect data from the hospital system with national death registration data. We provide an analysis covering all teaching and general hospitals in Scotland in a method that could be (where facilities and laws allow) replicated elsewhere. We have not yet been able to establish predictors of death within 12 months that relate to clinical or health indicators, such as diagnosis, co-morbidities or previous use of services.

The study is designed as a prevalent cohort study. There are certain implications to this design. One aspect is that patients with longer hospital stays will have a larger chance of becoming part of the ‘sample’ (though we would regard ours more properly as a ‘population’). Indeed, the probability of being sampled is proportional to length of stay (also known as length-biased sampling). This means that the results are not meaningful for individual patients, though of course that is not the stated interest of this article. But we must acknowledge that our results are likely to be different from a study where sampling was done on day of admission to hospital (an incident sample). Our study is intended to inform system-level debate about care of the hospital population in relation to end-of-life issues. An incident sample would inform thinking about the assessment of patients for end-of-life care needs, as they came into the hospital. Our sampling may also have influenced the relation between deprivation and mortality – with the possibility that more deprived patients are likely to be hospitalised longer.

## Conclusion

We have shown in the Scottish context that almost 1 in 10 patients in teaching or general hospitals at any given time will die during that admission. Almost 1 in 3 patients will have died a year later, rising to nearly 1 in 2 for the oldest groups. Hospitals are clearly an important context for end-of-life care, yet there are still difficulties in making the transition to palliative care and in implementing interventions for the imminently dying. In order that appropriate care plans can be made and delivered for patients, there is a strong need for hospitals to adopt a more vigorous approach to identifying patients who are entering the last years of their lives. We contend that the culture and organisation of hospitals need to become more attuned to the high proportion of inpatients in imminent need of end-of-life care.
